# Effect of Methylprednisolone on Inflammation and Coagulation in Patients with Severe COVID-19: A Retrospective Cohort Study

**DOI:** 10.1177/11772719211021647

**Published:** 2021-06-04

**Authors:** Katja Tromp, Philip van der Zee, Casper Rokx, Jeroen van Kampen, Diederik Gommers, Henrik Endeman

**Affiliations:** 1 Department of Adult Intensive Care, Erasmus Medical Center, Rotterdam, The Netherlands; 2 Department of Internal Medicine, section Infectious Diseases, Erasmus Medical Center, Rotterdam, The Netherlands; 3 Department of Viroscience, Erasmus Medical Center, Rotterdam, The Netherlands

**Keywords:** COVID-19, acute respiratory distress syndrome, corticosteroids, inflammation, coagulation, mechanical ventilation

## Abstract

Corticosteroids reduced mortality rate in patients with coronavirus disease 2019 (COVID-19). Previously, we hypothesized that corticosteroids mitigate the inflammation response resulting in reduced coagulation and thrombosis. In this retrospective study, we included 27 patients with COVID-19 that received high-dose corticosteroids (methylprednisolone 1000 mg i.v. daily for 3 days) for persistent respiratory failure or an excessive inflammation response. We found that inflammation, coagulation, and ventilation parameters improved significantly after methylprednisolone. The viral loads of SARS-CoV-2 remained stable or decreased. These results provides insight into the reduced mortality rate observed in patients with COVID-19 treated with corticosteroids.

**To the editor**

Corticosteroids reduced mortality rate in patients with coronavirus disease 2019 (COVID-19).^1^ Previously, we hypothesized that the inflammation response plays a role in the development of coagulation and thrombosis.^2^ In this retrospective study, we assessed the effects of corticosteroids on inflammation, coagulation, and ventilation parameters in mechanically ventilated patients with COVID-19. All patients with established COVID-19 admitted to the intensive care unit of the Erasmus MC that received high-dose methylprednisolone (1000 mg i.v. daily for 3 days) between February and May 2020 were included.

Methylprednisolone was indicated if a patient had persistent respiratory failure or an excessive inflammation response. Persistent respiratory failure was defined as progressive disease on CT scan at day 10 of mechanical ventilation as compared to CT scan at admission, in combination with either a respiratory system compliance <30 mL/cmH_2_O or a PaO_2_/FiO_2_ ratio <200 mmHg. Excessive inflammation response was defined as a modified H-score above 169 as described by Mehta et al^3^ in combination with progressive multi-organ failure. Prior to methylprednisolone, patients were screened for fungal or bacterial colonization and secondary infections in bronchoalveolar lavage fluid and blood cultures. After methylprednisolone, patients received prednisolone 1 mg/kg daily for 7 days and a tapering schedule according to the attending physician’s preferences. All patients received prophylactic anticoagulation (nadroparin 5700IU BID) or therapeutic anticoagulation (heparin pump) in case of established thrombotic complications. Patients received selective digestive decontamination as standard of care.^4^

Twenty-seven of the 127 patients with COVID-19 admitted to our ICU received methylprednisolone and were included in this study. Patient characteristics are shown in Table 1.

**Table 1.** Baseline characteristics.

Baseline	n = 27
Male-no. (%)	21 (81)
Age (years)	61 (46-70)
Body mass index	26.6 (25.4-31.4)
Medical history-no. (%)	
Cardiac	3 (12)
Hypertension	9 (35)
Diabetes mellitus	5 (19)
Neurological	1 (4)
Malignancy	2 (8)
Pulmonary	5 (19)
Deep vein thrombosis or pulmonary embolism	1 (4)
Other*	8 (31)
No medical history	4 (15)
Immunocompetent prior to admission	27 (100)
APACHE IV score	64 (53-84)
Days in ICU before methylprednisolone	13 (10-21)
Days of mechanical ventilation before methylprednisolone	13 (9-21)
Indication for methylprednisolone	
Persistent respiratory failure	19 (70.4)
Excessive inflammation response	8 (29.6)
Pulmonary embolism-no. (%)	14 (54)
SARS-CoV-2 IgM in serum-no. (%)	
Detectable	24 (89)
Non-detectable	2 (8)
Not determined	1 (4)
SARS-CoV-2 Ig in serum-no. (%)	
Detectable	25 (93)
Non-detectable	1 (4)
Not determined	1 (4)
28-day mortality-no. (%)	12 (44)
ICU length of stay (days)	28 (19-40)
Survivors	35 (28-42)
Non-survivors	21 (12-29)
Days on mechanical ventilation	27 (18-37)
Survivors	32 (21-39)
Non-survivors	21 (10-29)
Secondary infections**	
Catheter related blood stream infection	
Definite-no. (%)	2 (7)
Causative microorganism	S. epidermidis, E. faecium
Possible-no. (%)	1 (4)
Causative microorganism	S. epidermidis
Ventilator associated pneumonia-no. (%)	1 (4)
Causative microorganism	P. aeruginosa
Invasive pulmonary aspergillosis-no. (%)	0 (0)

All values are presented as median (IQR) unless stated otherwise.

Abbreviations: APACHE IV score, acute physiology and chronic health evaluation IV score; FiO_2_, fraction of inspired oxygen; ICU, intensive care unit; PaO_2_, partial pressure of arterial oxygen.

* Includes alcohol abuse, diaphragmatic hernia, hay fever, hypothyroidism, irritable bowel disease, morbid obesity, and radius fracture.

** Catheter related blood stream infection and ventilator-associated pneumonia were defined according to the clinical practice guidelines by the Infectious Diseases Society of America, and invasive pulmonary aspergillosis was defined according to the European Organization for Research and Treatment of Cancer/Invasive Fungal Infections Cooperative Group and the National Institute of Allergy and Infectious Diseases Mycoses Study Group Consensus Group.

The inflammation, coagulation, and ventilation parameters over time are shown in Table 2. We observed a significant decrease in leukocytes, lymphocytes, neutrophils, and interleukin-6 from Day 0 to Day 1. This decrease persisted up to Day 3 for lymphocytes and interleukin-6. C-reactive protein and procalcitonin decreased from Day 2 and Day 3, respectively, and remained low. D-dimers decreased on Day 1 and fibrinogen decreased on Day 2, both remained low. We observed an initial increase in thrombocytes on Day 2 and Day 3, and a subsequent decrease of thrombocytes on Day 14. The activated partial thromboplastin time (APTT) increased 15 seconds (*P* = .208) from Day 0 to Day 1, while the heparin dose initially remained similar and decreased significantly on Day 7. Three patients had supratherapeutic APTT (>80 seconds). No major bleeding or new thromboembolic events occurred.

**Table 2.** Inflammation, coagulation, and ventilation parameters after start of methylprednisolone.

	Day-1	Day 0	Day 1	Day 2	Day 3	Day 7	Day 14
Inflammation							
Leukocytes (10^9^/L)	12.8 (8.7-17.4)* (n = 23)	15.0 (10.4-18.6) (n = 25)	11.5 (8.2-16.4)* (n = 23)	11.8 (8.0-18.0) (n = 22)	11.6 (8.4-14.3) (n = 21)	16.0 (14.3-21.0) (n = 17)	14.8 (12.2-20.1) (n = 15)
Lymphocytes (%)	12.6 (11.0-15.0) (n = 21)	10.5 (7.7-13.8) (n = 23)	7.0 (5.3-11.3)* (n = 22)	7.2 (6.1-11.2)* (n = 22)	8.8 (5.8-11.8)* (n = 20)	12.0 (9.5-15.7) (n = 12)	14.7 (6.6-18.4) (n = 7)
Neutrophils (%)	73.6 (69.3-77.3) (n = 16)	74.8 (72.0-84.2) (n = 18)	86.4 (83.4-90.0)* (n = 17)	79.9 (75.2-83.4) (n = 15)	77.1 (70.2-81.0) (n = 13)	63.8 (62.2-73.1) (n = 9)	71.6 (65.8-80.5) (n = 6)
C-Reactive Protein (mg/L)	215 (156-279) (n = 23)	170 (105-259) (n = 25)	158 (105-174) (n = 23)	87 (48-152)* (n = 22)	57 (23-78)* (n = 21)	32 (25-53)* (n = 18)	35 (11-70)* (n = 15)
Procalcitonin (ng/mL)	2.0 (0.63-1.61) (n = 21)	1.03 (0.63-2.21) (n = 23)	1.28 (0.57-3.02) (n = 21)	0.76 (0.38-1.28) (n = 21)	0.59 (0.34-1.18)* (n = 20)	0.35 (0.25-1.06)* (n = 13)	0.17 (0.11-0.48)* (n = 7)
Ferritin (mg/L)	1607 (1169-2434) (n = 22)	1510 (1234-2664) (n = 24)	1397 (841-2509) (n = 22)	1330 (871-2468) (n = 22)	1109 (884-2831) (n = 20)	1114 (621-1866)* (n = 13)	1070 (727-1624) (n = 7)
Interleukin 6 (pg/mL)	79 (43-108) (n = 20)	103 (52-187) (n = 23)	10 (8-22)* (n = 19)	4 (3-7)* (n = 18)	5 (3-15)* (n = 20)	22 (15-32) (n = 12)	18 (9-24) (n = 6)
Coagulation							
Thrombocytes (10^9/L)	374 (298-452) (n = 23)	390 (323-476) (n = 25)	440 (290-491) (n = 22)	475 (335-512)* (n = 21)	458 (331-548)* (n = 21)	391 (291-451) (n = 17)	263 (190-368)* (n = 15)
D-dimers (mg/L)	2.79 (1.31-6.77) (n = 21)	3.78 (1.19-5.49) (n = 24)	2.46 (1.10-3.91)* (n = 22)	1.79 (0.96-3.13)* (n = 22)	1.75 (1.13-3.30)* (n = 20)	2.67 (1.50-4.86) (n = 13)	1.63 (1.26-2.85) (n = 7)
Fibrinogen (g/L)	7.0 (6.0-7.7) (n = 20)	7.1 (6.1-8.0) (n = 23)	7.2 (5.7-8.1) (n = 22)	5.5 (4.9-6.6)* (n = 22)	4.9 (4.2-6.3)* (n = 20)	5.0 (4.5-5.7)* (n = 13)	4.8 (4.3-6.5) (n = 7)
Anti-Xa-level (U/mL)	0.49 (0.29-0.60) (n = 14)	0.52 (0.41-0.65) (n = 15)	0.59 (0.43-0.71) (n = 16)	0.66 (0.27-0.84) (n = 14)	0.72 (0.40-0.94) (n = 14)	0.53 (0.34-0.65) (n = 13)	0.41 (0.38-0.55) (n = 10)
APTT (seconds)	36 (32-53) (n = 13)	35 (26-52) (n = 17)	50 (40-60) (n = 14)	40 (28-54) (n = 12)	33 (19-36) (n = 13)	33 (30-39) (n = 11)	28 (25-33) (n = 8)
Heparin (IU/day)	49200 (29125-59100) (n = 16)	50400 (34850-58200) (n = 17)	49200 (36000-60000) (n = 15)	42600 (17800-55500) (n = 14)	40800 (30925-55800) (n = 12)	32200 (24600-45900)* (n = 12)	36100 (20100-44475) (n = 10)
Mechanical ventilation							
PaO_2_/FiO_2_Ratio (mm Hg)	125 (103-209) (n = 23)	150 (113-203) (n = 24)	161 (113-210) (n = 22)	175 (117-221) (n = 22)	178 (119-236)* (n = 21)	248 (196-312)* (n = 16)	255 (235-342)* (n = 11)
Positive end-expiratory pressure (cm H_2_O)	13 (11-16) (n = 23)	12 (10-16) (n = 24)	12 (10-16) (n = 22)	10 (8-15) (n = 22)	10 (9-15)* (n = 21)	10 (8-13)* (n = 16)	7 (7-11)* (n = 11)
Peak pressure (cm H_2_O)	29 (27-34) (n = 23)	31 (27-34) (n = 24)	32 (26-34) (n = 22)	32 (25-36) (n = 22)	28 (26-34) (n = 21)	25 (20-30)* (n = 16)	18 (13-22)* (n = 11)
Compliance, respiratory system (mL/cm H_2_O)	30.0 (22.8-38.0) (n = 14)	27.5 (21.8-40.0) (n = 10)	28.0 (22.0-55.0) (n = 11)	29.0 (25.3-40.5) (n = 10)	30.0 (26.3-37.8) (n = 10)	46.0 (36.0-66.0)* (n = 10)	65.5 (42.8-75.8) (n = 8)
Minute volume (L/min)	14.5 (11.5-16.3)* (n = 23)	15.9 (12.1-19.1) (n = 24)	13.9 (12.2-17.3)* (n = 22)	14.7 (11.8-17.0) (n = 22)	13.6 (11.8-17.3)* (n = 21)	14.9 (11.6-16.6) (n = 16)	14.8 (12.4-16.3) (n = 11)
End-tidal CO_2_ (kPa)	4.9 (4.4-5.5) (n = 18)	4.7 (3.9-5.8) (n = 23)	4.4 (3.8-5.1) (n = 21)	4.4 (4.0-5.0) (n = 20)	4.7 (3.9-5.9) (n = 18)	4.5 (4.1-5.0) (n = 15)	4.2 (3.9-4.5) (n = 10)
PaCO_2_ (kPa)	6.1 (5.8-6.9) (n = 23)	6.4 (5.3-7.5) (n = 25)	6.3 (5.6-7.3) (n = 23)	6.2 (5.5-6.6) (n = 22)	6.2 (5.4-7.1) (n = 21)	5.8 (5.0-6.2) (n = 18)	5.3 (4.7-5.6)* (n = 15)
ΔCO_2_ (kPa)	1.8 (0.9-4.8) (n = 20)	1.5 (.9-2.6) (n = 24)	1.6 (1.3-2.8)* (n = 23)	1.6 (1.1-2.2) (n = 22)	1.7 (0.9-1.7) (n = 18)	1.3 (0.9-1.7) (n = 18)	1.4 (0.9-4.3) (n = 15)

Abbreviations: ΔCO_2_ CO_2_ gap PaCo_2_, minus End-Tidal CO_2_; FiO_2_, fraction of inspired oxygen; PaCO_2_, partial pressure of arterial carbon dioxide; PaO_2_, partial pressure of arterial oxygen.

* Statistically significant as compared to Day 0 (*P* < .05), tested with the Wilcoxon signed rank test. Data are presented as median (interquartile range). Day 0 is the start of methylprednisolone, Day-1 the day before start of treatment, Day 1-14 the days after start of treatment.

We observed a significant increase in PaO_2_/FiO_2_ ratio and a decrease in positive end-expiratory pressure (PEEP) from Day 3 up to Day 14. At Day 7, peak pressure was reduced and respiratory system compliance improved significantly.

The viral loads of SARS-CoV-2 generally remained stable or decreased after methylprednisolone. We observed 8 blood stream infections per 1000 catheter days, which was above the norm in our general ICU population of 3 infections per 1000 catheter days. One case of ventilator-associated pneumonia was observed. All infections occurred in different patients.

In this study, we showed that corticosteroids reduced inflammation and coagulation parameters after a median of 13 days of mechanical ventilation in patients with COVID-19. These data suggest that methylprednisolone is associated with reduced hypercoagulation, although it remains unclear whether corticosteroids reduce hypercoagulation directly, indirectly through reducing inflammatory mediators, or both. In accordance with previous research, this study suggests that high-dose corticosteroids did not result in increased replication of SARS-CoV-2 in a relatively late stage of COVID-19.^5^

Despite the small sample size, lack of control group, and the relatively late stage of COVID-19, this study showed that inflammation, coagulation, and ventilation parameters improved in patients with COVID-19 and provides us with greater insight into the reduced mortality rate observed in patients with COVID-19 treated with corticosteroids.
